# Posttraumatic Stress Disorder as a Consequence of Acute Cardiovascular Disease

**DOI:** 10.1007/s11886-023-01870-1

**Published:** 2023-05-02

**Authors:** Mary Princip, Katharina Ledermann, Roland von Känel

**Affiliations:** 1 Department of Consultation-Liaison Psychiatry and Psychosomatic Medicine, University Hospital Zurich, University of Zurich, Zurich, Switzerland; 2 Department of Clinical and Health Psychology, University of Fribourg, Fribourg, Switzerland

**Keywords:** Posttraumatic stress disorder (PTSD), Cardiovascular disease (CVD), Cardiac-induced posttraumatic stress disorder (CDI-PTSD), Acute coronary syndrome (ACS), Posttraumatic stress symptoms (PTSS)

## Abstract

**Purpose of Review:**

To provide an update of the current evidence of cardiac disease–induced posttraumatic stress disorder (CDI-PTSD) with a focus on acute coronary events.

**Recent Findings:**

A cardiovascular disease, particularly a life-threatening cardiac event is often a highly stressful experience that can induce PTSD in patients and their caregivers, taking a chronic course if left untreated. There are several features distinguishing CDI-PTSD from “traditional” PTSD induced by external trauma, namely enduring somatic threat, inability to avoid trauma-related cues and hyperarousal with internal body sensations leading to constant fear of recurrent cardiac events. An increased risk of recurrent CVD events may be explained by pathophysiological changes, an unhealthy lifestyle and non-adherence to cardiac treatment. A trauma-focused approach might be useful to treat CDI-PTSD.

**Summary:**

Treatment options for patients and caregivers as well as long-term effects of trauma-focused interventions on physical and mental health outcomes should be future research directions.

## Background and Introduction

Cardiovascular diseases (CVD), including acute cardiac events such as acute coronary syndrome (ACS), continue to be the major cause of morbidity and mortality worldwide [1]. In the USA, CVD are responsible for about one-third of all deaths and result in millions of hospital admissions annually [2]. Patients in low- and middle-income countries are particularly affected by CVD [3]. During the past several years, the rates of hospitalization and mortality due to CVD have decreased due to better medical care and risk factor control. Nevertheless, CVD place a major burden on health care systems with considerable medical and economical costs [4]. The sudden onset of an acute cardiac event, the actual risk of dying, and the perceived loss of control and helplessness during the event represent a potentially traumatic situation. This may contribute to the development of clinically relevant symptoms or a formal diagnosis of an acute stress disorder (ASD), which by definition occurs less than 1 month after a cardiac event, and/or a posttraumatic stress disorder (PTSD) [5]. The latter is defined in terms of the presence of several clusters of psychological, behavioral, and physiological symptoms, including intrusive thoughts, avoidance, negative alterations in cognitions and mood, increased arousal, and stress reactivity [6]. Cardiac disease–induced (CDI)-PTSD not only significantly impairs quality of life but is also associated with a 53% increased risk of another cardiac event and increased mortality in the first 3 years after the initial event [7, 8]. A similar association was also revealed for a twofold increased risk of incident stroke [9]. Taken together, this literature justifies why it is important to study the characteristics and peculiarities of CDI-PTSD to highlight the relevance of this clinical problem and to ensure accurate diagnosis and treatment.

### Prevalence

Table 1 shows the prevalence of clinically significant PTSD symptoms and a clinical diagnosis of PTSD induced by different types of CVD events and procedures. The majority of these epidemiological studies to date have focused on ACS as a potentially traumatic cardiac event [10]. An ACS describes the range of myocardial ischemic states that includes unstable angina, non-ST-elevated myocardial infarction (NSTEMI) or ST-elevated myocardial infarction (STEMI) [11]. During ACS, 22% of patients report intense distress and fear of dying, while 52% report moderate fear and distress. As a result, about 70% of patients feel anxious, depressed, or detached from their environment/themselves for a short time or are subject to strong emotional responses after ACS [12]. In the longer term, clinically relevant PTSD symptoms (i.e., subsyndromal PTSD) is found in 12% (95% CI), while 4% (95% CI) meet full criteria for PTSD based on a clinical/psychiatric interview [13]. Nearly one-third of patients persist to have PTSD up to 2 years after ACS [9]. In addition to studies in patients with ACS, there is a growing body of research showing that other forms of CVD and CVD-related procedures can also induce PTSD [14]. Particularly 20% of patients with an implantable cardioverter defibrillator (ICD) were found to have PTSD at their initial assessment [15]. These rates decreased significantly to 12% in the first 6 months after implantation and remained stable at 13% within 1 year [16]. Despite the initial reduction in the number of PTSD cases after implantation, another study found an increase in PTSD symptoms between 2 years and 5.5 years after implantation [17]. In addition, ICD patients with more severe PTSD symptoms were significantly more likely to experience shocks after device implantation [18]. In turn, five or more shocks as a threshold seems to be a risk factor for the development of elevated PTSD symptoms [17].

**Table 1:** Prevalence of clinically relevant PTSD symptoms as a consequence of CVD

**Diagnosis**	**Prevalence rates**	**Author and year**
Acute coronary syndrome	4–12%	Edmondson et al. 2012 [19]
Stroke and transcient ischemic attack	Overall 13% among stroke /TIA survivors Within 1 year: 23% 1 year: 11%	Edmondson et al. 2013 [13]
Surviving out-of-hospital cardiac arrest	20–38%; in caregivers ~ 35%	Yaow et al. 2022 [26]; Ladwig et al. 2008 [15]; Agarwal et al. 2022 [24]; Presciutti et al. 2021 [25]
Coronary artery bypass graft surgery	8–44%	Rawashdeh 2020 [20] Singh et al., 2017 [31]
Heart transplantation	13.5%	Loh et al. 2020 [32]
Ventricular assist device	None of the patients, but 23% of the partners	Weerahandi et al. 2017 [21]
Takotsubo syndrome	No prevalence rates available, but one case report showed PTSD in the aftermath of TTS	Herb et al. 2015 [22]
Spontaneous coronary artery dissection	28%	Johnson et al. 2022 [23]
Implantable cardioverter defibrillator	20%	Sears et al. 2011 [16]
Congenital heart disease	12–31%; in caregivers 30%	Meentken et al. 2017 [30]

Also, patients and/or their caregivers can develop PTSD after stroke/transient ischemic attack (TIA) [19], heart surgery, e.g., coronary artery bypass surgery or heart transplantation [20], implantation of an ventricular assist device (VAD) [21], stress cardiomyopathy/Takotsubo syndrome (TTS) [22], spontaneous coronary artery dissection (SCAD) [23], and cardiac arrest [15, 24–26]. Moreover, venous thromboembolism, including pulmonary embolism, can also be experienced as traumatic and subsequently lead to PTSD [27]. Therefore, it is important for clinicians to consider PTSD across a range of different CVD. It should be noted, however, that most of the studies mentioned here used self-report questionnaires that were still based on DSM-IV criteria [28]. In addition, CDI-PTSD has some unique clinical features that are inadequately captured by the commonly used instruments [29].

A topic that has received little attention in the literature is PTSD in children after cardiac surgery. A recent review [30] indicates that 12–31% of children undergoing cardiac surgery develop clinical PTSD and an additional 12–14% show clinically relevant PTSD symptoms. Screening for PTSD and offering psychological care for children and caregivers is necessary to prevent far-reaching consequences. However, whether this is routinely done in clinical practice has not systematically been investigated so far.

Another topic that has received little attention is caregivers of CVD event survivors, who are often forgotten in the discussions about CDI-PTSD. However, the significance of families as part of the survivorship journey has recently been acknowledged given that a cardiac arrest of a family member is a life-defining crisis for both the patient and the family [33]. The comparison of prevalence in PTSD between patients with VAD or congenital heart disease and their caregivers, as examples, clearly show that traumatic consequences of a cardiac disease regarding PTSD symptoms can even be more profound in caregivers than in patients. Roughly half of family members experience long-term symptoms such as mood disorders and PTSD symptoms [34]. The negative consequences of these events can possibly be limited through early counseling and follow-up. Existing resuscitation guidelines encourage important components of a family-centered approach such as advanced care planning, deliberate communication, evidence-based resuscitation initiation, and termination and family presence as an option [35]. A patient- and family-centered cardiac arrest care conceptual model focusing on care needs of families of families experiencing both fatal and non-fatal cardiac arrest has been suggested by Douma et al. [36••]. The model’s five components are (i) focus on the family member in cardiac arrest, (ii) collaboration of the resuscitation team and family, (iii) consideration of family context, (iv) family post-resuscitation needs, and (v) dedicated policies and procedures [36••]. New research findings in this regard would be highly desirable, specifically in relation to intervention strategies, rehabilitation, and future research directions and also in relation to the evaluation of the effect of family-centered cardiac care.

### DSM-5 Symptom Criteria for PTSD

**Fig. 1:**
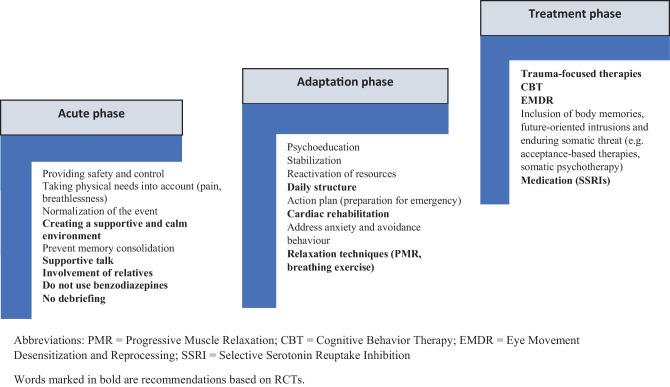
Implications for the treatment of CDI-PTSD. Words marked in bold are recommendations based on RCTs. PMR, progressive muscle relaxation; CBT, cognitive behavior therapy; EMDR, eye movement desensitization and reprocessing; SSRI, selective serotonin reuptake inhibition

PTSD is listed under “Trauma and Stress Disorders” in the Diagnostic and Statistical Manual of Mental disorders fifth edition (DSM-5) (309.81) (Fig. 1) [6]. Cardiac event/disease-induced PTSD is triggered by exposure to a heart disease, acute cardiac event, cardiac intervention (or its consequences) as a confrontation with actual or threatened death or serious injury (criterion A). Patients with PTSD present four distinct clusters of symptoms consisting of intrusive symptoms of the traumatic event (e.g., unwanted disturbing memories or nightmares; Criterion B), avoidance of memories of traumatic stimuli (Criterion C), negative changes in cognition and mood, decreased interest in activities (Criterion D), and hyperarousal (Criterion E). The disorder persists for more than 1 month (Criterion F) and causes clinically meaningful distress or impairment in social, occupational, or other important areas of functioning (Criterion G). Symptoms are not related to medications, substance use, or other illnesses (Criterion H). A distinction is made between acute PTSD (symptoms last less than 3 months), chronic PTSD (symptoms last at least 3 months) and PTSD with delayed onset (onset 6 months after the traumatic event) [6].

Individuals with CDI-PTSD (and/or their families) present with PTSD symptoms similar to those of individuals who have been exposed to other types of traumata. However, several distinguishing features exist [29]. Table 2 shows the differences and similarities between a traditional trauma and a CDI trauma.

**Table 2:** Differences and similarities between traditional and CDI trauma according to Edmondson et al. [29]

**Traditional trauma**	**Cardiac disease–induced trauma**
External cause	Internal cause (body, e.g., heart region)
Temporary threat	Persistent threat
Fear of dying	Fear of dying
Intrusions are oriented to the past (flash-back)	Intrusions are often future-oriented (flash-forward)
Avoidance behavior is possible	Avoidance behavior is difficult to carry out
Overexcitation is associated with external triggers	Overexcitation is associated with internal (body-related) triggers

### Differences Between Traditional and CDI Trauma

Acute cardiac events differ from other trauma in that the nature of the threat relates primarily to future-oriented intrusive thoughts. Therefore, the clinical presentation of CDI-PTSD differs in many ways from traditional PTSD. Characteristic of CDI-PTSD is internal self-inflicted threat that is persistent. In this context, Edmondson [29] proposed the enduric somatic threat (EST) model for medically induced PTSD, which considers the cardiac event or disease as permanent physical threat. In contrast, “traditional” trauma as a result of, e.g., rape, earthquake, experiencing war, has an external source of causation. Intrusion symptoms in CDI-PTSD can be experienced in the present or as future-oriented intrusions and may include thoughts, images, and sensations such as fear of being victimized or eventual cardiac death [29]. These future-oriented intrusions culminate in so-called flash-forward intrusions, which, unlike flashbacks, may include thoughts and images related to the threat of another cardiac event, as well as fear of dying or apprehension about upcoming physical examinations. Another difference concerns avoidance behavior, which is associated with negative health consequences for the patients. Because patients consciously avoid important health-promoting activities like physical exercise, seeing a physician, taking medication and participating in cardiac rehabilitation, they may develop negative feelings such as guilt and fear. Physical sensations such as increased heart rate, shortness of breath, and accelerated breathing are difficult to avoid and lead to misinterpretation of physical signals as an impending sign of further damage to the heart. Hyperarousal in CDI-PTSD is often associated with internal body sensations such as physical sensations related to the activity of the heart, whereas in traditional PTSD, triggers are often external. In CDI-PTSD, it can be challenging for both patients and clinicians to differentiate physical symptoms such as shortness of breath, chest pain, and dizziness as hyperarousal-related symptoms from “true” symptoms of the cardiac diseases, which may lead to constant fear of another cardiac event. Ironically, this vicious cycle of avoidance of physical activity and fear of another event may increase the risk of recurrence at various levels through different “cardiotoxic” effects. These include sleep disorders, increased blood pressure, lower heart rate variability, and inflammatory processes with detrimental cardiovascular consequences. It has not systematically been investigated if and to what extent the specifics of ACS-induced PTSD symptoms differ from those induced by for instance stroke or cardiac surgery. It may be conceivable that after a stroke, patients may also fear recurrence, while avoidance behavior may more closely relate to impairments in activities of daily living than in patients after ACS. The development of PTSD symptom questionnaires which consider these specific issues may be a fruitful subject of further research to inform tailored interventions.

### Screening for and Diagnosing PTSD in Cardiac Patients

PTSD that develops following a cardiac event is often not recognized or recognized late because of the particular symptom presentation, including future-related intrusions. Specific diagnostic tools for CDI-PTSD symptoms do not exist (see previous paragraph). Based on the recommendations of several guidelines and scientific statements for clinical practice, patients with a CVD should be screened for psychological stress [36••]. In order to specifically screen for PTSD in cardiac patients, the primary care checklist for PTSD (PC-PTSD-5) [37] can be used. The PC-PTSD-5 is a screen designed to identify individuals with probable PTSD. Those screening positive require further assessment, preferably with a structured interview. Table 3 shows a selection of instruments to assess CDI-PTSD with the clinician-administered PTSD scale for DSM-5 (CAPS-5) as the gold standard [38].

**Table 3:** Measures of PTSD symptoms following cardiac events

**Measures**	**Items (range)**	**Cut-offs**	**Assessment time**
**Screening instrument**			
Primary care checklist for PTSD (PC-PTSD-5) [37]	5 (yes or no)	For men, a cut-off of 4; for women, the cut-off should be lower due to a high number of false negatives	5 min
**Clinical interviews**			
Clinician-administered PTSD scale for DSM-5 (CAPS 5) Weathers et al. [38]	30 (0–4)	At least one Criterion B symptom At least one Criterion C symptom At least two Criterion D symptoms At least two Criterion E symptoms Criterion F is met (disturbance has lasted one month) Criterion G is met (disturbance causes either clinically significant distress or functional impairment)	45 min
Structured clinical interview for DSM-5 (SCID)-PTSD module First et al. [39]	21	The SCID is not quantitatively scored; all diagnostic symptoms are coded as present, subthreshold, or absent	20 min
Child PTSD symptom scale for DSM-5 (CPSS-5) Foa et al. [40]	27 (0–4)		30 min
**Self-report questionnaires**			
Posttraumatic checklist (PCL-5) Weathers et al. [41]	20 (0–4)	34	15–20 min
Posttraumatic diagnostic scale (PDS) Foa et al. [42]	24 (0–4)	15 (together with 1 symptom of re-experiencing, 3 symptoms of avoidance, 2 symptoms of arousal) or 1–10 mild, 11–20 moderate, 21–35 moderate to severe and > 36 severe	15–20 min
Impact of event scale–revised (IES) Weiss and Marmar [43]	22 (0–4)	46	15–20 min
Child PTSD symptom scale for DSM-5 self-report (CPSS 5 SR) Foa et al. [40]	20 (0–4)	31	10 min

### Screening Questions from the PC-PTSD-5:

Have you experienced your heart condition/disease so badly that in the last month you:

1. Had nightmares about it or had to think about it when you did not want to?
2. Tried hard not to think about the heart disease/event or avoided situations that reminded you of the heart condition/disease?
3. Been constantly on guard, watchful, or easily startled?
4. Felt numb or detached from people, activities, or your surroundings?
5. Felt guilty or unable to stop blaming yourself or others for the heart condition/disease or any problems the heart condition/disease may have caused?

### Scoring of PC-PTSD-5

If a respondent indicates a cardiac trauma history, the patient is instructed to answer five additional yes/no questions (see above) about CDI-PTSD symptoms in the past month. The total score is between 0 and 5, according to the number of a “yes” response to each question. For men, a cut-off of 4 should be considered for further evaluation, whereas for women the cut-off should be lower due to a high number of false negatives [37]. As is the case with all instruments shown in Table 3, it should be noted that the PC-PTSD-5 with its proposed cut-offs has not been validated in patients with CVD, which can be problematic given the mentioned specifics of CDI-PTSD symptoms.

### Risk Factors for the Development of CDI-PTSD Symptoms

Because not all patients develop PTSD, researchers have made extensive efforts to identify factors that are associated with an increased risk [28]. Particularly important is the individual perception of illness of a cardiac event which has consistently been shown to have an impact on the development of CDI-PTSD symptoms [44]. Specifically, if a person reacts to ACS with severe pain, fear, and helplessness, the risk of developing PTSD is significantly increased [44]. In contrast, objective parameters of cardiac injury (e.g., troponin T level in ACS) show little correlation with PTSD symptoms [45]. Various other risk factors for the development of CDI-PTSD symptoms relate to the environment and treatment of the disease (e.g., a hectic hospital admission, treatment complications, statements made by staff, use of benzodiazepines) [46, 47•], sociodemographic factors (younger age, female sex) [44], comorbidities (depression, anxiety disorders, ASD) [48], personality factors (type D, neuroticism, hostility, alexithymia) [49, 50], and a patient’s biopsychosocial history (stressful life events, previous heart disease, and other somatic diseases) [51]. In addition, even patients with suspected ACS that are ultimately ruled out can show PTSD symptoms and have a comparable risk to develop PTSD related to the acute event as patients with confirmed ACS [52]. Regarding the trajectory of PTSD symptoms with suspected and confirmed ACS patients, about 87% of ACS patients can be classified in a resilient trajectory group with low PTSD symptoms within 1 year. A second group (10%) shows a chronic worsening course. Finally, only 3% can be classified into an acute-recovering trajectory group characterized by initially high PTSD symptoms that steadily decrease over the course of 1 year [53]. On the other hand, social support [54], resilience factors (including internal control beliefs, humor, and patience) [55], as well as repressive coping strategies immediately after the traumatic event have been shown to have a protective effect with regard to the development of CDI-PTSD symptoms [56].

### Mechanisms Linking PTSD with an Increased Risk of CVD

PTSD has long been associated with an increased risk for CVD; however, the bi-directional relationship between PTSD and CVD is not fully established yet [57]. A number of physiological mechanisms have been studied to explain the relationship between PTSD and CVD, including autonomic dysfunction, neuro-humeral disturbance (renin-angiotensin systems, RAS), neuroendocrine (HPA-axis function), inflammatory and metabolic changes and maladaptive health behaviors (for details see Selgiowski et al.) [58]. There may be several neurobiological underpinnings that play a role, compatible with the concept of PTSD as a disorder of altered emotional memory formation and/or extinction [59] and dysregulation of the threat and stress response [60]. These include altered function and connectivity of specific brain regions involved in emotion processing and cognition, namely the amygdala, insula, dorsal anterior cingulate, and ventral medial prefrontal cortex. These changes correspond with altered behavioral manifestations such as increased attentional bias to threat and exaggerated physiological, emotional, and behavioral responses to threat cues.

Recently, Krantz et al. [61] proposed two alternate approaches to conceptualize the association between PTSD and CVD. The first model views PTSD as a mental health disorder that elicits stress-related responses that are causal factors in the atherosclerotic process similar to prior models (e.g., [62, 63]). In this model, PTSD is associated with physiological (e.g., sympathetic nervous system, inflammatory, endocrine), emotional (e.g., depression, anxiety, affect), and behavioral changes (e.g., smoking, substance abuse, diet, and exercise (for more details, see Krantz et al.) [61] that are very similar to those associated with psychosocial stress or allostatic load. In contrast, the second model displays a “systemic disorder model” of PTSD as a systemic disorder with biological, as well as behavioral risk factor components that understands PTSD as cardiovascular risk factor. In this model, PTSD and co-occuring physical disorders are explained as being a direct result of the collection of systemic changes and the biological dysregulation intrinsic to PTSD, meaning that they are not seen as comorbidities or unrelated diseases [61]. However, further research is clearly needed to determine the validity and utility of these two approaches.

### Efficacy of Psychotherapeutic Procedures for CDI-PTSD

Due to the chronic nature of CDI-PTSD symptoms, effective treatment options are sorely needed. Various types of psychotherapy, including cognitive behavioral therapy (CBT), exposure therapy, and eye movement desensitization and reprocessing (EMDR) have been shown to be effective treatments for PTSD [64]. Importantly, trauma-focused therapies are superior to non-trauma-focused therapies in reducing PTSD symptom severity [65]. However, treatment options for PTSD symptoms that have been induced by CVD have insufficiently been studied. Immediately after ACS, one single session of psychological counseling with a trauma-specific content did not significantly reduce ACS-induced PTSD symptoms compared with a counseling session on dealing with psychosocial stress as an active control intervention [66]. However, patients with greater social support or longer participation in cardiac rehabilitation showed fewer ACS-induced PTSD symptoms in the trauma-specific group compared with the control group at 1-year follow-up [67]. Particularly, the benefit of a supportive social environment in the prevention and treatment of CDI-PTSD should be considered a subject for future research.

A systematic review of controlled treatment studies in patients with medically induced PTSD showed that only two studies and one pilot study have focused on CDI-PTSD so far [64]. One study examined PTSD symptoms in 42 cardiac surgery patients after 4 weeks of either eye movement desensitization and reprocessing (EMDR) or prolonged imaginal exposure therapy (IET) [68]. Consistent with meta-analytic data suggesting that EMDR is somewhat more effective than CBT in treating PTSD, EMDR performed significantly better in reducing PTSD symptoms but also in alleviating depressive symptoms and anxiety in patients after cardiac surgery [68].

The second controlled study examined the effectiveness of three to five sessions of IET in 60 patients with different diagnoses of CVD compared to a group of patients with ACS receiving 1–3 sessions of psychoeducation. The results of this study showed no significant improvements in PTSD symptoms in the overall sample, but a reduction in the subgroup of patients who had an unscheduled CVD event and high PTSD symptom scores at baseline. In addition, the therapy proved to be safe in that there were no relevant changes in blood pressure and heart rate during IET and similar rates of recurrent hospitalizations, events, and invasive procedures during follow-up in the IET group and the control group [69].

In a pilot study with 20 patients, EMDR resulted in a reduction of ICD shock-induced PTSD symptoms within 1 year. However, as this was an observational study without a control group, randomized controlled trials (RCTs) are needed to establish effective treatment options for this patient group but also others with CDI-PTSD symptoms [70]. It remains particularly unclear if and to what extent treatment of PTSD symptoms has to be specified to each cardiac condition/CVD. Other treatment options such as online therapies could also be valuable for the treatment of CDI-PTSD. Alexithymia, particularly a lack of “identifying feelings” has been associated with the development of CDI-PTSD [49]. Therefore, it may be worthwhile to put a greater focus on emotion regulation and strategies to improve emotion awareness in the treatment of CDI-PTSD. Resilience has also been related to CDI-PTSD [55], such that resource-oriented techniques could potentially lead to a reduction in PTSD symptoms. In addition, future research questions could address the long-term effects of trauma-focused treatments on physical health outcomes and psychological well-being in survivors of a life-threatening CVD event. A review of 44 studies concluded that interventions for traditional (i.e., not medically induced) PTSD might improve cardiovascular physiological outcomes, particularly cardiovascular reactivity to trauma cues [71], although additional methodologically rigorous studies are needed. In this context, trauma-focused treatments could reduce the risk of recurrent CVD events and premature mortality and contribute to increased quality of life in the long term.

### Implications for the Treatment of CDI-PTSD

To date, there is no evidence for the effectiveness of any specific early intervention to prevent the development of CDI-PTSD. However, immediately after the traumatic event, so-called compulsory debriefing should be avoided, in which patients are urged to undergo strong emotional activation, as this may increase the psychological stress in the longer term [72•]. Rather, pain reduction and physical needs should be the treatment focus. After initial stabilization has been achieved, trauma-specific procedures can be used to treat future-oriented intrusions.

Figure 1 provides an overview of treatment options at different stages after a CVD event. These recommendations are based on the literature including care team approaches and/or own clinical experience and need to be tested further in RCTs.

#### Drug Treatment of CDI-PTSD

Proven alterations in the neurotransmitter system justify the treatment of severe PTSD symptoms with psychopharmacological substances. Selective serotonin reuptake inhibitors (SSRIs) are considered the drugs of choice for PTSD treatment. At the same time, sertraline and citalopram are the only substances to date that can be considered safe in patients with CVD based on randomized placebo-controlled studies [73]. A note of caution has to be made for patients with heart failure in whom SSRIs are likely associated with an increased risk of mortality [74]. A note of caution has to be made for patients with heart failure in whom SSRIs are likely associated with an increased risk of mortality [75]. A recent meta-analysis has shown that antidepressants could increase the risk of all-cause mortality in patients with heart failure and that this effect is independent of whether patients have depression or the type of antidepressants used [76]. For example, the randomized, controlled MOOD-HF trial, included in this meta-analysis, showed that patients with depressive symptom scores in the moderate–severe depression range who received an SSRI had significantly higher all-cause mortality or hospitalizations after 2 years of follow-up than patients who received placebo. Based on the currently available evidence, a position paper endorsed by the European Association of Preventive Cardiology on mental health–related risk factors and interventions in patients with heart failure came to the conclusion that caution should be exercised in psychopharmacological interventions in patients with heart failure because they may increase all-cause mortality [74]. Tricyclics should be avoided in patients with CVD. The use of other psychotropic drugs is based on the guidelines for the therapy of PTSD that has not been induced by a CVD event, taking into account, however, cardiometabolic side effects (e.g., weight gain with olanzapine and mirtazapine). Regular ECG and laboratory monitoring is also recommended for SSRI therapy, since QT interval prolongation (e.g., with citalopram > 40 mg daily) can occur with the risk of dangerous cardiac arrhythmias. There are currently no RCTs on therapeutic drugs for the specific treatment of CDI-PTSD.

### Further Directions of Research

Further research into CDI-PTSD is paramount to supporting affected patients and containing medical and economic costs. Although important differences from traditional PTSD have already been identified, it seems important to further investigate, monitor, and address CDI-PTSD symptoms to identify and support affected patients at an early stage. In this context, a specific diagnostic instrument would be desirable, which can reliably be applied to screen for possible CDI-PTSD symptoms. Subsequently, specific therapeutic procedures should also be developed, the efficacy of which can be tested in RCTs. Intervention strategies that also address avoidance behavior of CDI-PTSD patients, such as lack of regular physical activities or irregular intake of medications, should be taken into account. These strategies might improve the cardiac prognosis. Specific mechanisms underlying treatment success should be tested, e.g., fear habituation and improved self-efficacy. It seems also important to focus research on protective factors such as implementation of social support, adaptive coping strategies, resilience, and hedonic well-being/optimism to possibly prevent the development of CDI-PTSD. Furthermore, care services for families and caregivers of patients following an acute cardiac event (e.g., ACS, cardiac arrest) should be established. In addition, future research could examine the long-term effects of trauma-focused interventions on physical and mental health outcomes in survivors of life-threatening CVD events.

## Conclusions

CDI-PTSD differs from “traditional PTSD” in that intrusions are often future-oriented, which may lead to underdiagnosis. In addition to impact psychological well-being, CDI-PTSD is associated with an increased risk of adverse CVD outcomes and increased mortality. There are several plausible biobehavioral mechanisms to explain this risk. CBT, including EMDR, and SSRIs have been shown to be effective treatments for PTSD, which appear particularly appropriate for patients with CVD. However, evidence from RCTs is still lacking for firm recommendations. Treatment may focus on restoring confidence in cardiac function by processing traumatic memories and reducing avoidance and safety behaviors to improve quality of life, daily functioning, and possibly cardiovascular prognosis.
